# IL-22-mediates Cross-talk between Tumor Cells and Immune Cells Associated with Favorable Prognosis in Human Colorectal Cancer

**DOI:** 10.33696/immunology.3.087

**Published:** 2021

**Authors:** Raoul André Droeser, Giandomenica Iezzi

**Affiliations:** 1 University Center for Gastrointestinal and Liver Diseases, Clarunis, University of Basel, Basel, Switzerland; 2 Department of Surgery, Ente Ospedaliero Cantonale and Università Svizzera Italiana, Lugano, Switzerland

**Keywords:** Human colorectal cancer, IL-22, Neutrophils, Tissue microarray, Prognosis, Chemokines

## Abstract

The positive prognostic role of the immune environment in colorectal cancer is widely accepted. However, there are few data about the prognostic significance of interleukin-22 in human colorectal cancer which is still debated. In our study we could demonstrate for the first time a positive prognostic role of interleukin-22 in human colorectal cancer relying on its capacity to induce in tumor cells the production of chemokines recruiting into the tumor microenvironment neutrophils associated with a favorable clinical outcome.

Colorectal cancer (CRC) is the third most common cause of cancer related death worldwide [1]. Its outcome depends on different factors. On one hand there are cancer related features, including mutations, microsatellite status, and methylation alterations. In addition, the tumor microenvironment, which includes non-transformed stromal and tumor infiltrating cells, interacting with cancer cells, also significantly influences tumor biology and consequently patients’ survival [2]. Infiltration by cellular components of the adaptive immune system, and, in particular, by cytotoxic CD8+ and T-helper type 1 lymphocytes, has been shown to predict the survival of patients with CRC more efficiently than the tumor-node-metastasis (TNM) staging [3].

In previous studies we could demonstrate that also cells of the innate immune system, such as neutrophilic granulocytes are associated with a favorable clinical outcome, possibly due to their co-stimulatory activity on CD8+ T cells [4]. However, the role of immune cells producing interleukin-22 (IL-22) in CRC progression remains a matter of debate.

IL-22 can be produced by a variety of immune cells, including conventional T lymphocytes and innate lymphoid cells [5]. Instead, the IL-22 receptor, including IL-22Rα and IL-10Rβ chains, is uniquely expressed on non-hematopoietic cells, including keratinocytes and intestinal epithelial cells [6]. IL-22 mediates pleiotropic functions. In different anatomical districts, such as skin and intestinal and bronchial epithelium, IL-22 synergizes with IL-17 and TNFα to promote host defense [7–9] and innate immunity to bacterial infections. On the other hand, IL-22 induces epithelial cell proliferation and up-regulation of genes encoding pro-survival molecules [10–13] and may protect liver, intestine and lung from tissue destruction [11–16]. Interestingly, IL-22 also plays a role in the maintenance of host-microbiota symbiosis [17].

Studies published so far, mainly based on murine models, support a tumor promoting role of IL-22 in hepatocellular carcinoma [18,19] and liposarcoma [20]. IL-22 production has also been shown to promote CRC development [21], possibly by direct effects on stem cells [22] or by enhancing cancer cell proliferation [23,24]. Most recently, however, IL-22 has been shown to play a key role in the control of genotoxic damage induced by carcinogens in colon epithelial stem cells, thereby limiting mutagenesis and cancer outgrowth [25]. Thus, in the intestine IL-22 might function as a double-edged sword, as on the one hand it mediates protective functions, through induction of epithelial regeneration and production of antimicrobial peptides, and on the other, it supports tumorigenesis, by increasing proliferation and apoptotic resistance of neoplastic epithelial cells [6,24]. Notably, most studies are based on mouse models of colitis-associated CRC, whereas in humans the majority of CRC develop in the absence of inflammatory conditions. On the other hand, there is paucity of data based on human studies. A specific polymorphism in IL-22 gene is associated with increased risk of developing CRC [26]. Furthermore, IL-22 synergizes with IFN-γ to induce iNOS production in human colon carcinoma cell lines with consequent production of pro-carcinogenic nitric oxygen species [27]. However, the overall impact of IL-22 on clinical outcome of CRC in humans has not been investigated so far.

We have evaluated the prognostic significance of IL-22 in human primary CRC, using two independent tissue microarrays (TMA), cumulatively including 514 samples. We observed that infiltration by IL-22 immune cells is significantly associated with an increased 5-year survival rate, independently of known prognostic factors including age, sex, T stage, N stage, tumor grade, vascular invasion, tumor border configuration, and microsatellite stability.

IL-22-producing cells, were detectable within normal colonic tissues and CRC, although, in the latter case, at a significantly higher density. *Ex vivo* phenotypic analysis revealed that they mainly consist of conventional T-helper cells (Th22), with a large majority also expressing IFN-γ and IL-17.

Interestingly, when we evaluated the direct effects of IL-22 on CRC cells lines, we did not observe any significant effect on cell proliferation. In contrast, IL-22 treatment consistently increased in CRC cells the expression of CXCL1, CXCL2 and CXCL3 neutrophil recruiting chemokines at gene and protein level. Furthermore, culture supernatants from IL-22-exposed CRC cells enhanced neutrophil migration *in vitro.*

Consistent with the *in vitro* data, a significant correlation between expression of IL-22 and that of CXCL_1_, CXCL_2_ and CXCL_3_ genes was observed in 597 CRC specimens included in the TCGA database [28].

To the best of our knowledge, we demonstrated for the first time a positive prognostic impact of tumor infiltrating Th22 cells in human CRC. This beneficial effect relies on the capacity of Th22 cells to enhance tumor infiltration by neutrophilic granulocytes by favoring release of neutrophil recruiting chemokines from CRC cells (Figure 1).

Taken together our data delineate a complex cross-talk occurring between tumor cells, T lymphocytes and neutrophil granulocytes within the CRC microenvironment. Further studies are warranted to clarify the role of additional components of CRC microenvironment, including in particular gut microbiota, potentially contributing to Th22 recruitment [29] and activation, and to neutrophil functional modulation.

## Figures and Tables

**Figure 1:**
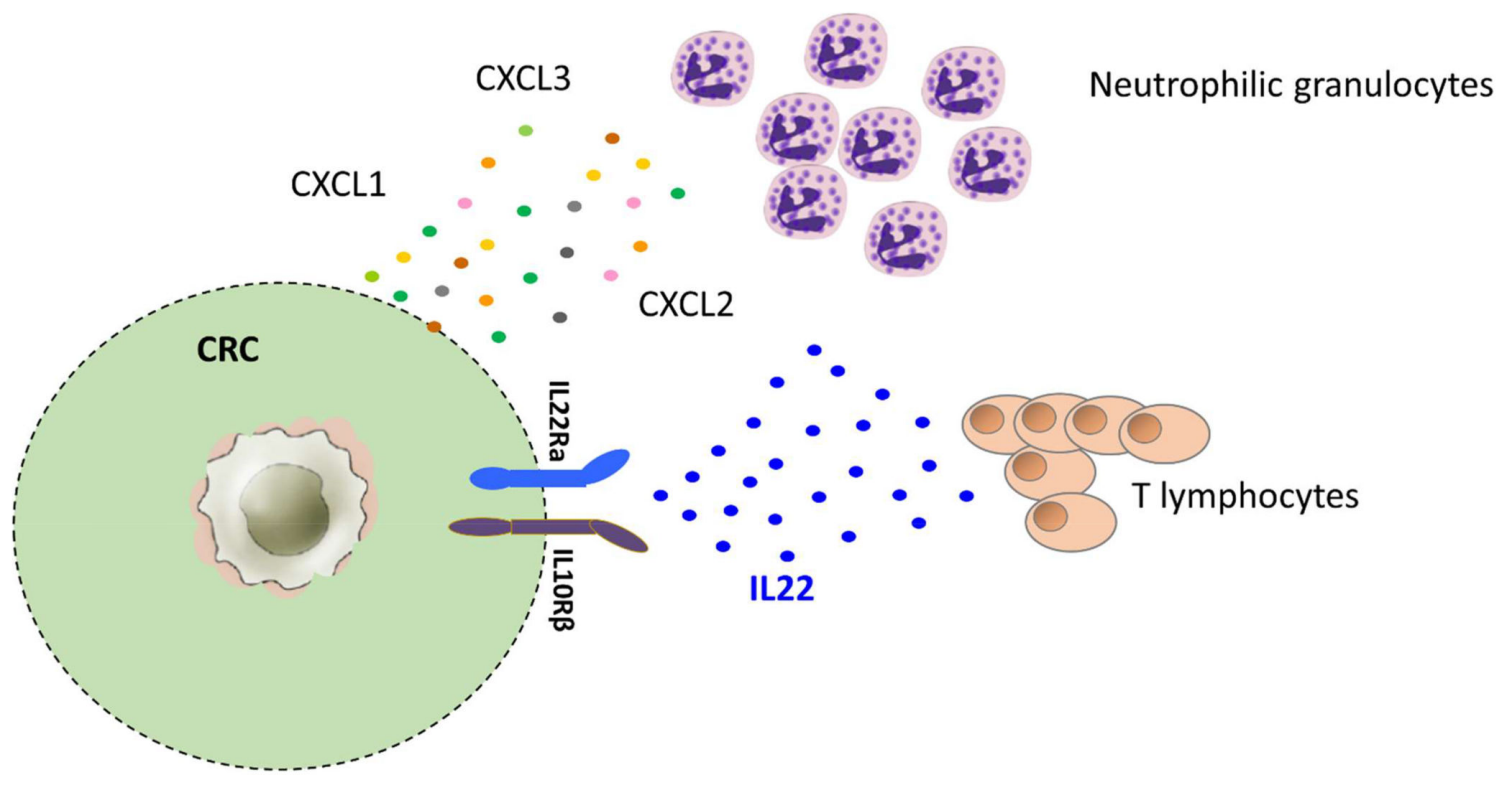
Mechanism underlying the favorable prognostic effect of IL-22-producing cells in human CRC. IL-22-producing T lymphocytes stimulate CRC cells to secrete CXCL_1_, CXCL_2_ and CXCL_3_ chemokines ultimately recruiting neutrophils endowed with anti-tumor functions into CRC tissues.
